# Providing clinicians with a patient’s 10-year cardiovascular risk improves their statin prescribing: a true experiment using clinical vignettes

**DOI:** 10.1186/1471-2261-13-90

**Published:** 2013-10-22

**Authors:** Nishant K Sekaran, Jeremy B Sussman, Anna Xu, Rodney A Hayward

**Affiliations:** 1 Division of General Internal Medicine, University of Michigan Medical School & VA Ann Arbor Healthcare System, 3119 Taubman Center, 1500 East Medical Center Drive, 48109-5604 Ann Arbor, MI, USA; 2 Division of General Internal Medicine, VA Ann Arbor Healthcare System & the Department of Internal Medicine, University of Michigan, Ann Arbor, MI, USA; 3 Michigan Institute for Healthcare Policy & Innovation, VA Ann Arbor Healthcare System & the Department of Internal Medicine, University of Michigan, Ann Arbor, MI, USA

**Keywords:** Primary prevention, Cardiovascular disease, Statins, Cardiovascular risk

## Abstract

**Background:**

Statins are effective for primary prevention of cardiovascular (CV) disease, the leading cause of death in the world. Multinational guidelines emphasize CV risk as an important factor for optimal statin prescribing. However, it’s not clear how primary care providers (PCPs) use this information. The objective of this study was to determine how primary care providers use information about global CV risk for primary prevention of CV disease.

**Methods:**

A double-blinded, randomized experiment using clinical vignettes mailed to office-based PCPs in the United States who were identified through the American Medical Association Physician Masterfile in June 2012. PCPs in the control group received clinical vignettes with all information on the risk factors needed to calculate CV risk. The experimental group received the same vignettes in addition to the subject’s 10-year calculated CV risk (Framingham risk score). The primary study outcome was the decision to prescribe a statin.

**Results:**

Providing calculated CV risk to providers increased statin prescribing in the two high-risk cases (CV risk > 20%) by 32 percentage points (41% v. 73%; 95% CI = 23-40, p <0.001; relative risk [RR] = 1.78) and 16 percentage points (12% v. 27%, 95% CI 8.5-22.5%, p <0.001; RR = 2.25), and decreased statin prescribing in the lowest risk case (CV risk = 2% risk) by 9 percentage points [95% CI = 1.00-16.7%, p = 0.003, RR = 0.88]. Fewer than 20% of participants in each group reported routinely calculating 10-year CV risk in their patients.

**Conclusions:**

Providers do not routinely calculate 10-year CV risk for their patients. In this vignette experiment, PCPs undertreated low LDL, high CV risk patients. Giving providers a patient’s calculated CV risk improved statin prescribing. Providing PCPs with accurate estimates of patient CV risk at the point of service has the potential to improve the efficiency of statin prescribing.

## Background

Cardiovascular (CV) disease is the leading cause of death in the world, with coronary artery disease alone resulting in over 7 million deaths annually [1]. HMG-CoA reductase inhibitors, or statins, are among the most effective and widely used treatments for reducing CV morbidity and mortality. Although there are substantial differences between recommendations for when and how to use statins for primary CV prevention, virtually all multinational clinical guidelines recommend estimating a patient’s overall CV risk in clinical decision making [2,3].

Although past guidelines for primary CV prevention in the U.S., including the ATP III NCEP III guideline, (3) have focused on specific lipid targets (i.e., a “treat to target” approach), there is increasing evidence for and interest in basing treatment primarily on overall CV risk [4]. Even ATP III guidelines base the individual patient’s recommended low-density lipoprotein cholesterol (LDL-C-C) goal on their overall CV risk, and it is likely that CV risk will be even more central in the soon-to-be released ATP IV guidelines. This is because recent evidence demonstrates how “tailoring” statin treatment based directly on the patient’s overall CV risk and expected benefit from a given statin dose can be a much more efficient approach than using LDL-C targets, preventing many more CV events while using less medication [5]. Although the greater efficacy of this type of benefit-based tailored (BTT) approach has gone almost completely unopposed scientifically, some critics have suggested that an approach so heavily based on CV risk is too complicated to be effectively implemented [6].

Current research suggests providers believe knowing a patient’s overall CV risk is important [7]. However, in routine practice most providers do not regularly calculate CV risk, estimate risk inaccurately, and tend to ignore important variations in CV risk [8-13]. Two potential reasons for this are 1) clinicians believe they can “gestalt” a patient’s CV risk, and 2) the amount of time it would take clinicians to do the calculation is a barrier. In the era of the electronic medical record, it is possible to automate estimates of patient’s overall CV risk for the clinician, as is now often done for the kidney glomerular filtration rate (GFR), and efficiently integrate this information into clinical workflow.

There has been limited research on the effect of CV risk presentation on primary care physicians’ (PCPs) primary CV prevention practices [14,15]. These studies suggest that explicit CV risk presentation may modestly improve provider prescription practices with regards to lipid lowering therapy. To our knowledge, none of these studies addressed the issue of how providers respond to clinical situations where LDL-C measurements are discordant from and are a poor reflection of a patient’s true overall CV risk. Knowing how PCPs reconcile this dilemma is important because it provides needed insight about how clinicians prioritize CV risk in statin treatment decisions, and how a BTT approach to primary CV prevention may be effectively implemented.

We examined this issue in a true experiment using clinical vignettes, in which we randomized primary care providers (PCPs) into two groups: a control group that received clinical vignettes of patients with information on their individual CV risk factors, and an experimental group that received the identical cases in addition to individual 10-year CV risk as determined by the Framingham Risk Score [16], a validated tool to estimate cardiovascular risk. We designed the clinical vignettes to include a wide range of 10-year CV risk, and specifically included cases where LDL-C values were normal but clinical risk was moderate to high (situations in which some guidelines would recommend no medication treatment). We hypothesized that explicit presentation of the overall CV risk calculation would improve the statin prescribing efficiency among PCPs—they would preferentially prescribe statins to higher expected benefit rather than lower benefit patients.

## Methods

### Experimental design

We used a double-blinded, randomized, controlled experimental design. All study subjects were provided with an identical set of clinical scenarios, which included all the information needed to calculate 10-year CV risk, with the additional information of 10-year Framingham CV risk estimates for individuals randomized to the experimental arm.

### Setting and participants

We mailed surveys to a random sample of 1,500 office-based adult primary care physicians (PCP) trained in Internal Medicine or Family Medicine practicing in the United States. We identified these providers through random sampling of the American Medical Association (AMA) Physician Masterfile, which contains practice information from 153,675 office-based internal medicine and family physicians in the United States. We obtained the data through the Direct Medical Data (DMD) Corporation (Des Plaines, IL), after receiving approval from The University of Michigan Institutional Review Board. DMD randomly selected and assigned the study subjects into an experimental or a control arm and provided the research team with de-identified data files.

### Survey instrument and intervention

The survey consisted of several background questions and 5 clinical vignettes, each containing a brief clinical history, including all CV risk factors in the Framingham risk score reported by D’Agostino and colleagues [16]. The clinical cases were designed to capture a range of predicted CV risk and LDL-C values. We pilot tested the surveys for clarity and content in a convenience sample of physicians at our institution. The complete surveys can be found in Additional file 1 (Control Group Survey) and Additional file 2 (Experimental Group Survey). Study subjects received a $20 bill as a monetary incentive. We sent a reminder postcard to survey respondents one week prior to the requested due date. The data from the returned surveys were abstracted and double-entered into the study database.

### Outcomes

Our primary outcome was the likelihood of prescribing a statin for each clinical vignette for the two treatment arms (after a trial of diet and exercise), measured on a five-point Likert Scale. Secondary outcomes included estimation of 10-year CV risk among respondents in the control group, PCPs self-reported target LDL-C goals, and several questions about their clinical practice.

### Statistical methods

We used a 2-sample independent groups *t*-test for our main analysis comparing the likelihood of prescribing a statin between our control and experimental arms for each vignette. We used a 1-sample *t*-test to compare predicted control respondents’ estimates of vignette subjects’ 10-year CV risk, comparing their estimate to the calculated Framingham risk scores. Our sample size of 626 respondents (313 individuals in each arm) provides us with 80% power to detect a difference of 10 percentage points between the control and experimental groups (0.2 v. 0.3, two-sided testing with alpha = 0.05).

After conducting the main *a priori* analyses, we performed a single post-hoc analysis of how a control PCP’s estimate of the patient’s 10-year CV risk impacted the probability that they would prescribe a statin for that case. All statistical analyses were performed using Stata 12 (College Station, TX).

## Results

### Characteristics of survey respondents

**Figure 1:**
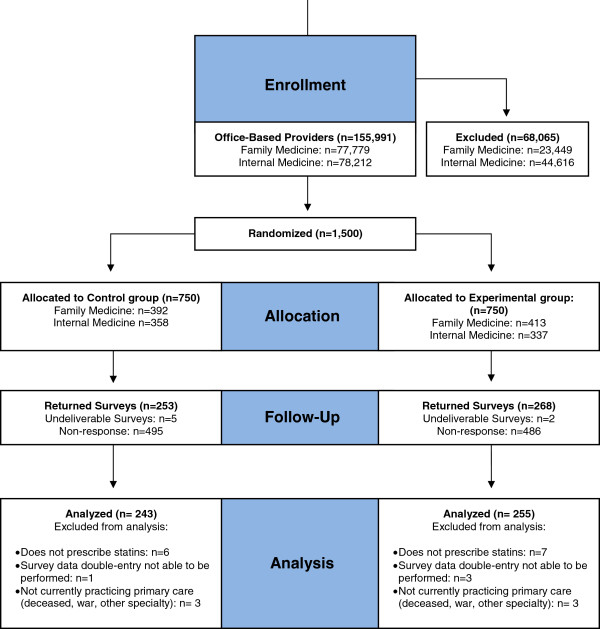
Flow of respondents through the randomized experiment.

Of the 1500 PCPs sent surveys, a total of 520 responded (35%). Figure 1 illustrates the flow of respondents. Table 1 displays their characteristics and shows that the control and intervention groups are well matched across a range of attributes. Approximately two-thirds of the survey respondents were family medicine trained, nearly 75% were in practice for 10 or more years, and the majority saw more than 40 patients per week (nearly half seeing more than 80 patients).

**Table 1:** Characteristics of survey respondents in the analysis sample

**Respondent characteristics**	**Control group (n = 243)**	**Experimental group (n = 255)**
Specialty		
Family medicine	62.3	61.9
Internal medicine	37.7	38.1
Post-graduate practice years in an outpatient setting		
<5 years	5.9	6.4
5-10	17.0	16.3
>10	77.1	77.3
Number of patients seen/week		
< 40	10.0	13.4
40-59	14.9	10.9
60-79	23.6	21.5
>80	51.5	54.3
Practice region		
Northeast	18.2	17.1
Midwest	30.6	26.6
South	31	30.6
West	20.3	25.8
How often do you predict 10-year cardiovascular risk in your patients?		
Almost never or never	49.8	43.7
Sometimes	31.1	41.9
Almost always or always	19.1	14.4
I would allow a trained nurse or member of my support staff to address the issue of primary prevention for coronary artery disease with my patients through an approved standard protocol?		
Strongly disagree or disagree	13.6	15.2
Neither agree nor disagree	19.5	17.9
Agree or strongly agree	67.0	67.0

### Estimation of 10-year cardiovascular (CV) risk

A minority of respondents reported regularly calculating 10-year CV risk (<20% in each group). Control group PCP estimates for each vignette’s 10-year CV risk are displayed in Table 2. For the low CV risk vignette patients (vignettes 1 and 3), PCPs overestimated CV risk by more than 9 percentage points (both *P* < 0.001). For example, Case #1′s 10-yr CV risk was only 2%, but over 75% of respondents estimated her risk as being 9.5% or greater. In contrast, for the two highest CV risk vignette patients (Cases #2 and #5, CV risks 21% and 22%, respectively), PCP mean estimates were significantly underestimated, but were within 5 percentage points of their Framingham score. The combined effect of these under- and over-estimations were striking, however. For example, although Case # 2's 10-yr CV risk (21%) was 3-times higher that Case #3 (7%), PCP estimates of these two vignette’s CV risk were almost identical: 19.3% (interquartile range [IQR] = 17.3%-21.3%) and 19.7 (IQR = 17.5-21.9), respectively.

**Table 2:** Clinical scenarios and associated 10-year CVD risk estimates

**Clinical scenario (n = number responding to the question in the control group)**	**Calculated 10-year Framingham risk for coronary artery disease**	Mean predicted 10-year cardiovascular risk and interquartile range among respondents in the control group* (N = 243)	**p-value for differences between calculated and mean predicted 10-year CVD risk**
Case 1. A 52 year-old woman with no chronic conditions, no history of tobacco or family history of cardiovascular disease; blood pressure 128/82, pulse 72, BMI 30; after a trial of diet and exercise, total cholesterol 260 mg/dL, triglycerides 145 mg/dL, HDL 56 mg/dL, and LDL-C 175 mg/dL (n = 235)	2	11.2 [9.5-12.8]	<0.001
Case 2. A 70 year-old man with hypertension, treated with a thiazide, and osteoarthritis. He quit tobacco 40 years ago; blood pressure 136/80, pulse 70; after a trial of diet and exercise, total cholesterol 208 mg/dL, triglycerides 190 mg/dL, HDL 42 mg/dL, and LDL-C 128 mg/dL (n = 232).	22	19.3 [17.3-21.3]	0.007
Case 3. A 55 year-old woman with hypertension, treated with a calcium channel blocker, and obesity; she smokes 1 pack of cigarettes daily, and has no family history of cardiovascular disease or diabetes; blood pressure 128/82, pulse 72, BMI 32; after a trial of diet and exercise, total choleseterol is 200 mg/dL, triglycerides 125 mg/dL, HDL 40 mg/dL, and LDL-C 135 mg/dL (n = 233).	7	19.7 [17.5-21.9]	<0.001
Case 4. A 52 year-old man with hypertension, treated with a thiazide; he smokes 1 pack of cigarettes daily, and has no family history of cardiovascular disease; blood pressure is 128/82, pulse 72; after a trial of diet and exercise, total cholesterol is 145 mg/dL, triglycerides 125 mg/dL, HDL 30 mg/dL, and LDL-C 90 mg/dL (n = 231)	13	16.7 [14.9-18.5]	<0.001
Case 5. A 71 year-old man with hypertension, treated with an ace inhibitor, and benign prostatic hyperplasia; he quit tobacco 30 years ago; blood pressure 136/80, pulse 70; total cholesterol 178 mg/dL, triglycerides 190 mg/dL, HDL 44 mg/dL, and LDL-C 96 mg/dL (n = 228)	21	15.9 [14.3-17.5]	<0.001

*Mean predicted 10-year CVD risk based on one-sample *t*-test.

### Impact of providing patient CV risk to PCPs on statin prescribing

The control and experimental groups both prescribed statins at the highest rates for the two patient vignettes with the highest LDL-C values but the lowest overall CV-risk (cases #1 and #3). In each of these cases, PCPs in both arms prescribed statins >60% of the time. Further, being informed that the 10-yr CV risk was low had only a small, though statistically significant, 9 percentage point absolute decrease in the likelihood of prescribing a statin in both cases (*P* = 0.03 and *P* = 0.04, Table 3). In contrast, for patients with relatively low LDL-C values but with the highest CV-risks (Cases # 2 and 5, 10-yr risks of 21% and 22%, respectively), providing PCPs with overall CV risk estimates substantially increased statin prescribing. In Case #2 (a 70 year old man with a 21% 10-year CV-risk), the proportion of PCPs prescribing a statin increased from 41% to 73% with presentation of risk (increase = 32 points [95% CI = 23-40]). Overall, congruence with NCEP III recommendations were moderate to high for Cases #4 & #5, but a majority of PCPs diverged from NCEP III guideline recommendations by recommending treatment in a low-risk woman with an LDL of 135 (Case #3, see Table 3).

**Table 3:** Physician recommendations for statin treatment by intervention group compared to NCEP III guidelines and a tailored treatment approach

	**ATP III NCEP III statin treatment recommendation**	**Benefit based therapy statin treatment recommendation**	**Proportion of control group prescribing statin therapy**	**Proportion of experimental group prescribing statin therapy**	Mean difference* [control -experimental groups [95% CI]	***P *** **value**
Case 1. 52 year-old woman: 10-year risk, 2%; LDL-C (175 mg/dL)	Optional	No statin	0.76	0.67	0.09 [0.007, 0.167]	0.03
Case 2. 70 year-old man male: 10-year risk, 22%; LDL-C (128 mg/dL)	Optional	High-potency statin	0.41	0.73	-0.32 [-0.397, -0.231]	<0.001
Case 3. 55 year-old woman: 10-year risk, 7%; LDL-C (135 mg/dL)	No	Moderate-potency statin	0.74	0.65	0.09 [0.003, 0.166]	0.04
Case 4. 52 year-old man: 10-year risk, 13%; LDL-C (90 mg/dL)	No	Moderate-potency statin	0.20	0.26	-0.06 [-0.136, 0.132]	0.11
Case 5. 71 year-old man: 10-year risk, 21%; LDL-C (96 mg/dL)	No	High-potency statin	0.12	0.27	-0.16 [-0.225, -0.085]	<0.001

^*^ Mean differences between control and experimental groups are representative as +/- numbers. Positive numbers favor statin treatment among respondents in the control group, and negative numbers favor statin treatment among respondents in the intervention group.

**Figure 2:**
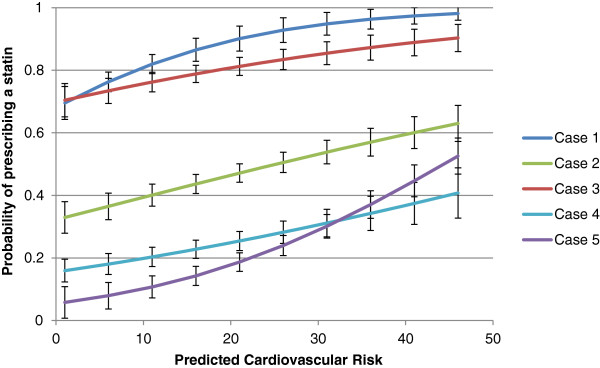
Primary care providers’ probability of prescribing a statin based on their perception of estimated CV risk.

In a *post hoc* analysis of respondents in the control arm (who were not given the calculated Framingham score), we found that the higher a PCP’s estimate of 10-year CV risk, the greater the likelihood they would prescribe a statin. This means that those control physicians who prescribed a statin in Cases #1 and #3 (high LDL-C/low CV risk/low statin benefit) were those who most severely over-estimated 10-year CV risk, and that those control physicians who did not prescribe a statin in Cases #2 and #5 (low LDL-C/high CV risk/high statin benefit) were those who most severely under-estimated 10-year CV risk (Figure 2).

## Discussion

Preventing cardiovascular (CV) disease morbidity and mortality is among the most important public health priorities in the United States and throughout the world, and statins are highly effective for primary prevention [17,18]. Selecting who should receive them, and at what doses, have been the subject of various guidelines and considerable debate. One consistent fact is that randomized clinical trial evidence suggests that overall CV risk is by far the largest determinant of how much absolute benefit a patient will receive from a statin [5]. In this experiment, we found providing PCPs with overall CV risk in clinical vignettes substantially decreased underuse of statins in high CV risk cases with “normal” LDL-C, patients for whom there has been grade A evidence for high benefit from a statin for the past decade [19]. We found that PCPs substantially under-estimated the CV risk of these low LDL-C patient vignettes, and this was associated with substantial under-prescribing.

We also found that PCP’s prescribed statins at the highest rates for the case with the lowest CV risk (2%) and a high LDL-C. Whether this represents “over-treatment” is a matter of debate. While the evidence suggests such a patient is much less likely, compared to a higher risk patient, to have averted a CV event over a 5-10 year period, some lipid experts have argued for treatment based upon the concept of elevated “lifetime risk” [20]. This might in part explain our finding that providing PCPs with the overall CV risk for such a patient (low CV risk with elevated LDL-C) only modestly reduced statin prescribing in this case. Nonetheless, we did find that those control PCPs who most severely over-estimated CV risk in cases with high LDL-C were also the most likely to prescribe a statin.

Our findings are similar to other studies finding infrequent calculation and inaccurate estimation of overall CV risk [7-12]. While this alone was not surprising, the magnitude of statin treatment intensity in both the control and experimental groups for lower risk vignettes in the study (CV risk of 2% and 7%) were particularly striking in our national sample, contrasting with much lower statin prescribing rates recently reported by PCPs at a single academic center [15]. In addition, the relative increases in treatment intensity in the experimental group for the high risk vignettes (CV risk of 21% and 22%) were much higher than in other studies of risk presentation to PCPs ([15,21,22]. Our results are consistent with, but cannot prove, that providers over-value high and low LDL levels, resulting in over-estimating CV risk in high LDL subjects and under-estimating risk in low LDL patients.

The practice tendencies among PCPs in our study—of infrequent use and inaccurate estimation of overall CV risk, especially in light of the substantial under-treatment of cases with high CV risk and low LDL-C —are concerning. However, our study results suggest that these practice patterns are potentially modifiable and highlight opportunities for improvement. While we hope that our results and those of others will lead to more physicians calculating their patients’ CV risks (or having someone do it for them), we are entering a new era of the electronic medical record that offers even greater potential. Experience from point-of-service A1c results [23] and automated GFR estimates [24] demonstrate how care can be substantially improved by providing PCPs with valuable information at the point of service. Automating CV risk prediction in a modern EHR has the potential to improve CV prevention, not just regarding statin therapy, but for other CV preventive care as well [25].

There are certain limitations of our study that merit particular note. We used the Framingham risk score, an established cardiovascular risk score that has been widely validated and cited in the medical literature [16,26]. However, this score uses a limited set of clinical variables and may not capture all aspects of individual risk [27]. Providers should use the risk-prediction tool that has the best evidence for accuracy and reliability in their population [28]. Second, although common in physician studies, our study had a low response rate. However, as a true experiment, a low response rate is less likely to alter the experimental findings – that providing PCPs with patient CV risk estimates can reduce under-treatment. It could alter the generalizability of our descriptive findings, particularly the precise proportion of PCPs that calculate CV risk. Our study evaluates the effect of presenting 10-year CV risk information on PCP statin prescribing, especially in cases where LDL-C is at “target”, and is not designed to explain how or why changes occurred. Third, these vignettes represent hypothetical scenarios and the answers may not reflect providers’ actual practices. Previous studies have established the validity of clinical vignettes for assessing clinical decision-making [29,30]. These scenarios were selected for clinical importance. By using the same scenarios for every respondent, we improved study power.

This study raises important future research questions about clinical decision-making for primary CV prevention. More needs to be known about how providers understand and use information about clinical risk in their treatment decisions especially since evidence suggests they value this information. Our study showed that even after informing PCPs that a patient with an elevated LDL had a very low 10-year CV risk, most prescribed a statin. Further research can deconstruct the dynamics of this complex decision making process including provider attention to existing ATP III NCEP III (and soon to be published ATP IV) guidelines, knowledge about LDL-C and CV disease pathogenesis, familiarity with patient preferences for statin therapy, and the role of adverse risks associated with this medicine class.

## Conclusion

In summary, our study found that providing calculated CV risk information to PCPs improves their statin prescribing practices for patients at the lowest and highest CV risks. PCPs in this study were seldom explicit about overall CV risk in their treatment decisions, and were not able to reliably estimate CV risk. Their statin prescribing patterns make clear that CV risk is not the primary factor influencing their statin prescribing decisions. Our results suggest, but cannot prove, that clinicians preferentially “anchor” on LDL-C treatment targets even when they diverge from overall CV risk. Incorporating automated CV risk information into clinical workflow processes, similar to other point-of-service data, using health information technology is possible and consistent with current standards of “meaningful use” [31]. In addition, guidelines recommending risk-based approaches should be based on sound clinical evidence, and not include other elements that would distract from the goal of minimizing harm and maximizing therapeutic benefit in individuals at moderate to high-risk of CV disease.

## Competing interests

The authors declare that they have no competing interests.

## Authors’ contributions

Conception and Design: NS, JS, AX and RH. Acquisition of Data: NS, JS and AX. Analysis and Interpretation of Data: NS, JS, and RH. Manuscript Preparation and Revision: NS, JS, AX, and RH. All authors read and approved the final manuscript.

## Pre-publication history

The pre-publication history for this paper can be accessed here:

http://www.biomedcentral.com/1471-2261/13/90/prepub

## Supplementary Material

Additional file 1 Control Group Survey. Click here for file

Additional file 2: Experimental Group Survey. Click here for file
