# Supplementary material for: Providing clinicians with a patient’s 10-year cardiovascular risk improves their statin prescribing: a true experiment using clinical vignettes
Source: BMC Cardiovasc Disord. 2013 Oct 22;13:90. doi: 10.1186/1471-2261-13-90 (PMC3924357; doi:10.1186/1471-2261-13-90)
Supplement: Additional file 1 — Control Group Survey. [file 1471-2261-13-90-S1.pdf]

Case 5:

- A 71 year-old man with hypertension, treated with an ace inhibitor, and benign prostatic hyperplasia.
- He quit tobacco 30 years ago.
- Blood pressure is 136/80, pulse 70.
- Total cholesterol is 178 mg/dL, triglycerides 190 mg/dL, HDL 44 mg/dL, and LDL 96 mg/dL (after trial of diet and exercise).

1. I am likely to prescribe a statin.

1	2	3	4	5
Strongly Disagree	Disagree	Neither Agree nor Disagree	Agree	Strongly Agree

2. If you did start a statin in this patient, what would be your LDL target (market the answer that is closest)?

1	2	3	4	5
<70 mg/dL	<100 mg/dL	<130 mg/dL	<160 mg/dL	N/A

3. Without using a risk calculator, what is your best single estimate (nearest % point) of this patient's 10-year cardiovascular risk?

   Approximately a _____% 10-year risk of a cardiovascular event

Contact Information

Nishant K. Sekaran, M.D. M.Sc.
University of Michigan Medical School

University of Michigan Medical School Institutional Review Board (IRBMED)
2800 Plymouth Road
Building 200, Room 2086
Ann Arbor, MI 48109-2800
Telephone: 734-763-4768 Fax: 734-763-1234
e-mail: irbmed@umich.edu

Support provided by a grant from the Robert Wood Johnson Foundation Community Health Leaders Program

Please continue on the back

Part C: Decision Support

The following questions pertain to treatment decisions regarding primary prevention of coronary artery disease
Please circle your response below each statement

1. I calculate 10-year cardiovascular risk for my patients to assist with treatment planning.

1	2	3	4	5
Never	Almost Never	Sometimes	Almost always	Always

2. The following factors would motivate me to use (or continue to use) information about 10-year cardiovascular risk in my treatment planning:

   Please rank the following from
   1-4 in order of 1 (most important) to 4 (least important)

   ______ Better, high-quality clinical trial evidence about the risks and benefits of using this information

   ______ Treatment guidelines from my specialty society

   ______ A computerized decision support tool guiding me how to use this information

   ______ More education from my peers or local medical society about the risks and benefits of using this information

3. I would allow a trained nurse or other trained member of my clinical support staff to address the issue of primary prevention for coronary artery disease with my patients through the use of a standard protocol that I approve.

1	2	3	4	5
Strongly Disagree	Disagree	Neither Agree nor Disagree	Agree	Strongly Agree

Thank you for your participation!

☐ Do not prescribe statins

☐ Please exclude my responses to the vignettes

A 5-minute Cardiovascular Disease Prevention Survey

Your practice makes a difference for your patients

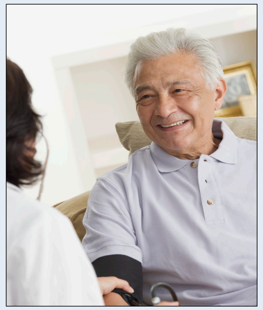
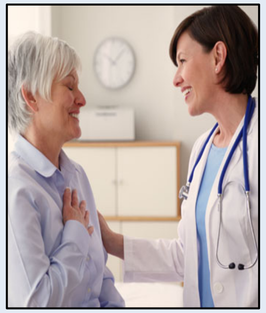
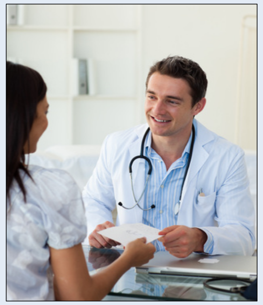
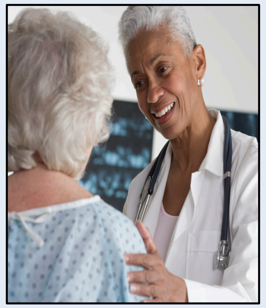

$20 enclosed
Please return by June 20, 2012

University of Michigan
Health System

Dear Colleague:

Our group of physician researchers from the University of Michigan (UM) is conducting a national survey examining statin prescription practices for coronary artery disease prevention in the adult ambulatory care setting. This study has been granted approval by the Institutional Review Board at UM.

We obtained your name from a random selection process of AMA Physician Masterfile. We are interested in clinical practice patterns of office-based internal medicine and family physicians in the United States.

Your participation in this survey is entirely voluntary, and your responses to the survey questions will be kept confidential. Your information will be stored anonymously and securely. Your responses indicate your consent to participate.

Please send your survey back in the self-addressed stamped envelope by June 20, 2012. <u>If you do not prescribe statins, please fill out Part A and mark the “do not prescribe” box on the back of the survey.</u> If you choose not to participate, please check the “exclude” box on the back of the survey. All survey recipients may keep the $20 bill as a token of our gratitude.

Part A: Your Background

Please check the appropriate box

1. What is your degree credential?
   - ☐ M.D or D.O Family Medicine
   - ☐ M.D. or D.O. Internal Medicine

2. Are you subspecialty trained?
   - ☐ No
   - ☐ If yes, what field? ____________________

3. How many years have you been practicing in an outpatient setting (after post-graduate training)?
   - ☐ <3 years
   - ☐ 3-5 years
   - ☐ 5-10 years
   - ☐ >10 years
   - ☐ N/A

4. How many outpatients (on average) do you see in a week?
   - ☐ <20 patients
   - ☐ 20-39 patients
   - ☐ 40-59 patients
   - ☐ 60-79 patients
   - ☐ >80 patients
   - ☐ N/A

Please continue to middle insert

Part B: Clinical Vignettes

We would like to assess **<u>your current clinical</u>** practice

Please circle your response below each statement

Case 1:

- A 52 year-old woman with no chronic medical conditions, no history of tobacco smoking or family history of cardiovascular disease.
- Blood pressure 128/82, pulse 72, BMI 30.
- Total cholesterol is 260 mg/dL, triglycerides 145 mg/dL, HDL 56 mg/dL, and LDL 175 mg/dL (after trial of diet and exercise).

1. I am likely to prescribe a statin.

1	2	3	4	5
Strongly Disagree	Disagree	Neither Agree nor Disagree	Agree	Strongly Agree

2. If you did start a statin in this patient, what would be your LDL target (mark the answer that is closest)?

1	2	3	4	5
<70 mg/dL	<100 mg/dL	<130 mg/dL	<160 mg/dL	N/A

3. Without using a risk calculator, what is your best <u>single estimate</u> (nearest % point) of this patient’s 10-year cardiovascular risk?

   Approximately a _____% 10-year risk of a cardiovascular event

Case 2:

- A 70 year-old man with hypertension, treated with a thiazide, and osteoarthritis. He quit tobacco 40 years ago.
- Blood pressure is 136/80, pulse 70.
- Total cholesterol is 208 mg/dL, triglycerides 190 mg/dL, HDL 42 mg/dL, and LDL 128 mg/dL (after trial of diet and exercise).

1. I am likely to prescribe a statin.

1	2	3	4	5
Strongly Disagree	Disagree	Neither Agree nor Disagree	Agree	Strongly Agree

2. If you did start a statin in this patient, what would be your LDL target (mark the answer that is closest)

1	2	3	4	5
<70 mg/dL	<100 mg/dL	<130 mg/dL	<160 mg/dL	N/A

3. Without using a risk calculator, what is your best <u>single estimate</u> (nearest % point) of this patient’s 10-year cardiovascular risk?

   Approximately a _____% 10-year risk of a cardiovascular event

Case 3:

- A 55 year-old woman with hypertension, treated with a calcium channel blocker, and obesity.
- She smokes 1 pack of cigarettes daily.
- No family history of cardiovascular disease or diabetes.
- Blood pressure is 128/82, pulse 72, BMI 32.
- Total cholesterol is 200 mg/dL, triglycerides 125 mg/dL, HDL 40 mg/dL, and LDL 135 mg/dL (after trial of diet and exercise).

1. I am likely to prescribe a statin.

1	2	3	4	5
Strongly Disagree	Disagree	Neither Agree nor Disagree	Agree	Strongly Agree

2. If you did start a statin in this patient, what would be your LDL target (mark the answer that is closest)?

1	2	3	4	5
<70 mg/dL	<100 mg/dL	<130 mg/dL	<160 mg/dL	N/A

3. Without using a risk calculator, what is your best <u>single estimate</u> (nearest % point) of this patient’s 10-year cardiovascular risk?

   Approximately a _____% 10-year risk of a cardiovascular event

Case 4:

- A 52 year-old man with hypertension, treated with a thiazide.
- He smokes 1 pack of cigarettes daily.
- No family history of cardiovascular disease.
- Blood pressure is 128/82, pulse 72.
- Total cholesterol is 145 mg/dL, triglycerides 125 mg/dL, HDL 30 mg/dL, and LDL 90 mg/dL (after trial of diet and exercise).

1. I am likely to prescribe a statin.

1	2	3	4	5
Strongly Disagree	Disagree	Neither Agree nor Disagree	Agree	Strongly Agree

2. If you did start a statin in this patient, what would be your LDL target (mark the answer that is closest)?

1	2	3	4	5
<70 mg/dL	<100 mg/dL	<130 mg/dL	<160 mg/dL	N/A

3. Without using a risk calculator, what is your best <u>single estimate</u> (nearest % point) of this patient’s 10-year cardiovascular risk?

   Approximately a _____% 10-year risk of a cardiovascular event

Please continue on the back
